# Supplementary material for: Complexome profiling of the Chlamydomonas psb28 mutant reveals TEF5 as an early PSII assembly factor
Source: Plant Cell. 2025 Mar 17;37(6):koaf055. doi: 10.1093/plcell/koaf055 (PMC12164590; doi:10.1093/plcell/koaf055)
Supplement: koaf055_Supplementary_Data [file koaf055_supplementary_data.zip › psb28_interactive.zip!/plots/intensity/qualityPlots/peptides/CAV9_Cre07.g333535.t1.1.html]

### Cre07.g333535.t1.1

Trivial name: CAV9  
SeqCoverage [median]: 0.70  
Mass: 298.25 kDa  
  
 

| Sequence | MissedCleavage | Mass [Da] | wtIntensity | muIntensity | Score |
| --- | --- | --- | --- | --- | --- |
| IGTGLLGAAQGRNSHLSIPSR | 1 | 2104.15 | 78.19 | 423.61 | 55.86 |
|  |  |  | 78.2 | 423.6 |  |
